# Jitter Detection and Image Restoration Based on Generative Adversarial Networks in Satellite Images

**DOI:** 10.3390/s21144693

**Published:** 2021-07-09

**Authors:** Zilin Wang, Zhaoxiang Zhang, Limin Dong, Guodong Xu

**Affiliations:** Research Center of Satellite Technology, Harbin Institute of Technology, Harbin 150001, China; zilin.wang@hit.edu.cn (Z.W.); zhaoxiangzxz@163.com (Z.Z.); donglimin@hit.edu.cn (L.D.)

**Keywords:** remote sensing, jitter detection, image restoration, convolutional neural network, generative adversarial network

## Abstract

High-resolution satellite images (HRSIs) obtained from onboard satellite linear array cameras suffer from geometric disturbance in the presence of attitude jitter. Therefore, detection and compensation of satellite attitude jitter are crucial to reduce the geopositioning error and to improve the geometric accuracy of HRSIs. In this work, a generative adversarial network (GAN) architecture is proposed to automatically learn and correct the deformed scene features from a single remote sensing image. In the proposed GAN, a convolutional neural network (CNN) is designed to discriminate the inputs, and another CNN is used to generate so-called fake inputs. To explore the usefulness and effectiveness of a GAN for jitter detection, the proposed GANs are trained on part of the PatternNet dataset and tested on three popular remote sensing datasets, along with a deformed Yaogan-26 satellite image. Several experiments show that the proposed model provides competitive results. The proposed GAN reveals the enormous potential of GAN-based methods for the analysis of attitude jitter from remote sensing images.

## 1. Introduction

In the application of high-resolution (HR) optical satellites, attitude jitter is a key factor affecting the accuracy of geopositioning and 3D mapping. Satellite jitter is commonly induced by the satellite’s thermal change, attitude control operation, dynamic structure and other factors [1,2,3]. Jitter is extremely difficult to eliminate [4]. Due to the complexity of jitter sources, satellite jitter is unavoidable, and its frequencies and amplitudes vary among satellites.

The linear array pushbroom camera has been used in high resolution remote sensing as a mature sensor device. Line scan imaging refers to forming a line image, or even a two-dimensional image. During each scan, the same scan line is imaged through the center projection, such as a linear CCD pushbroom camera [5]. Therefore, with respect to a satellite equipped with CCD linear array sensors, attitude jitter can deteriorate the geopositioning and mapping accuracy of HR satellites in both plane and height [6]. The warping of remote sensing images and the attitude variations are well known and are of wide concern.

**Figure 1:**
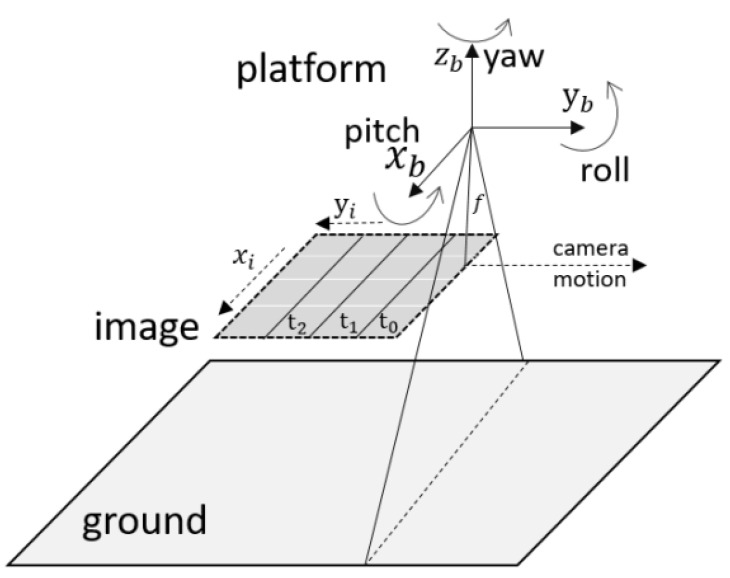
Overview of the pushbroom acquisition principle.

**Figure 2:**
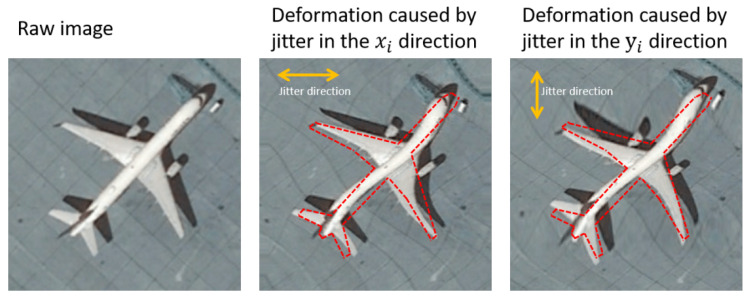
Image deformation caused by jitter in the $x_{i}$ and $y_{i}$ directions.

The oscillations of a spacecraft around its rotation axis consequently deform the geometric performance of HR satellite imagery. Remote sensing satellites, such as Terra, ALOS, MOMS-2P, and QuickBird, suffer from satellite jitter. Figure 1 shows the acquisition principle of the pushbroom camera from satellite platforms. The camera is moving straight along the $y_{b}$ axis and recording 1D images over time, denoted by t. Jitter in the pitch and row directions deforms the images in the $x_{i}$ and $y_{i}$ directions, respectively. According to existing research [7], the influence of jitter in the yaw direction is sufficiently small to be neglected. Figure 2 illustrates the deformed image caused by jitter in the two directions. Jitter in the $x_{i}$ direction twists the airplane horizontally, and jitter in the $y_{i}$ direction stretches and compresses the image in the vertical direction. In practice, image deformation occurs simultaneously in the two directions. Therefore, compensating images by means of classic image processing algorithms becomes difficult. Consequently, advanced image processing methods, such as deep learning, are required for this task.

Generally, three traditional methods are used to detect spacecraft jitter and restore deformed remote sensing images. The first is to use high-performance attitude sensors to obtain the attitude information with high time and angular resolution [8]. The second is to use ground control points (GCPs) in the remote sensing scenes [9,10]. Methods that depend on accurate GCPs or high-performance attitude sensors are economically and technically infeasible for many on-orbit satellites [6]. The third method is to take advantage of the fact that pushbroom sensors use the parallax formed by neighboring multispectral sensors [11,12,13,14,15,16,17]. The method requires image pairs collected at the same location in different times and has high requirements for the accuracy of feature extraction and image matching. Thus, new jitter detection and image compensation methods are required, providing only deformed panchromatic images when adjacent bands are not available.

In recent years, the study of feature extraction is particularly important. Fan, Mengbao, et al. proposed the phase of spectral PEC response to serve as an original feature in pulsed eddy current (PEC) research and presented a strategy to determine frequency and select superior feature [18]. Furthermore, many deep learning models, especially deep convolutional neural networks (CNNs), have been proposed in the remote sensing community. In [19], a novel pixel-pair method was proposed as a deep spectral classifier to classify remote sensing images with insufficient training samples. Zi et al. introduced a double-branch principal component analysis (PCA) network to segment cloud areas from remote sensing images [20]. Zhou et al. demonstrated the excellent performance of CNN methods on remote sensing image retrieval tasks [21]. Moreover, CNNs also show great potential in various image processing tasks, such as image denoising [22], pan sharpening [23], and target recognition [24].

Although considerable progress has been made, deep-learning-based models often encounter a serious problem known as overfitting when the training data are limited. Unfortunately, because data preparation is time-consuming and expensive, training data are commonly limited in the remote sensing community, which substantially restricts the generalization of models. Thus, effective training strategies for deep models are required to address the issue of overfitting. In a deep convolutional GAN model, the discriminator network of the GAN can be regarded as a regularization method that can force the generator network to learn essential features and mitigate overfitting to a great extent. Because of the advantages of deep convolutional GAN, GAN has shown great feasibility in a variety of remote sensing applications, such as image classification [25], image pan sharpening [26], and image translation [27].

In this work, the application of GANs for deformed image compensation is original introduced to explore for attitude correction. We have conducted a comprehensive and in-depth study on attitude jitter analysis and the restoration of deformed images based on the generative adversarial network, by using completely different ideas and methods to design a new network architecture that can automatically detect jitter and restore deformed remote sensing images end-to-end through one network architecture. A GAN-based image jitter compensation network (RestoreGAN) for remote sensing images is proposed. RestoreGAN takes a single deformed image as input and outputs a restored image along with jitter curves. The experimental results for three datasets and a Yaogan-26 deformed image illustrate the superiority of RestoreGAN compared with other methods under the condition of limited training samples. The rest of this work is organized as follows. Section 2 presents the background of satellite jitter and the details of the proposed GAN framework, including image interpretation architectures for jitter detection, along with the introduction of adversarial losses for image compensation. Details of the experimental results and discussions are reported in Section 3. In Section 4, conclusions are presented. The major contributions of the paper are summed up as follows.

1. Aiming at detection and compensation of satellite attitude jitter, in this paper, a generative adversarial network architecture is original introduced to automatically learn and correct the deformed scene features from a single remote sensing image suffer from geometric disturbance in the presence of attitude jitter.
2. We proposed a new GAN-based image jitter compensation network (RestoreGAN) for remote sensing images. Compared with the previous architecture, two convolution blocks with large kernels are first applied, which has been proven helpful to learn the HR features and improve network capability. Then, one stride convolution block and two residual blocks with batch ormalization are introduced. The discriminator network of the GAN can be regarded as a regularization method that can force the generator network to learn essential features and mitigate overfitting to a great extent.
3. We constructed a comprehensive and in-depth study on the analysis of attitude jitter from remote sensing images based on generative adversarial network. The experimental results on three public datasets indicate that the proposed framework achieves the highest DM and best performance on most of the restored images. The image retrieval results demonstrate the necessity and effectiveness of the proposed method in image retrieval tasks.

## 2. Materials and Methods

### 2.1. Jitter Displacement Estimation Modeling

A jitter detection and compensation model is established to compensate for the image distortion caused by satellite jitter. According to previous work [28], jitter displacement can be modeled as an attitude jitter component combined with one or more sinusoidal functions of different frequencies, amplitudes and phases, as demonstrated in Equation (1):

(1) $$\Delta \phi_{p}\left(t\right)=\sum_{i=1}^{N}A_{pi}\sin\left(2\pi f_{pi}+\phi_{pi}\right)$$

Here, $A_{pi}$ demotes the amplitude. $f_{pi}$ demotes the frequency. $\phi_{pi}$ denotes the phase of the sinusoidal function. $\Delta \phi_{p}\left(t\right)$ denotes the attitude jitter at imaging time *t*. Furthermore, *p* denotes the attitude jitter direction in the body coordinate reference system, as shown in the Figure 1. According to the jitter detection results for ZY-3 and Yaogan-26 satellites images [5,7], satellite jitter in the body coordinate system is composed of three parts: a sinusoidal curve with the dominant frequency and maximum amplitude, several high-frequency curves with small amplitude, and several low-frequency curves with small amplitude. Therefore, provided the satellite intrinsic parameters, Equation (1) can be rewritten as:

(2) $$\Delta \phi_{p}\left(t\right)=A_{m}\sin\left(2\pi f_{m}+\phi_{m}\right)+\sum_{i=1}^{N_{h}}A_{h}\sin\left(2\pi f_{h}+\phi_{h}\right)+\sum_{i=1}^{N_{l}}A_{l}\sin\left(2\pi f_{l}+\phi_{l}\right).$$

Here, the first term on the right-hand side of the equation is the dominant frequency sinusoidal function. The second and third terms are the high-frequency and low-frequency parts of the jitter displacement, respectively. In the satellite platform, the dominant frequency is based on the expected attitude accuracy of the AOCS control system, which is expected to have the largest influence on attitude inaccuracies [29]. The high-frequency jitter is caused by the vibration of the platform, wheels, CMGs, and flexible accessories and the transient motion of the moving parts. The low-frequency jitter is generated by temperature variation, seasonal variation, orbital perturbation, gravity gradient moments, and so on [30].

In the geometric compensation, the relationship between the satellite attitude jitter and the pixel displacement of the images is demonstrated below:

(3) $$D_{p}\left(x_{i}\right)=\frac{\Delta \phi_{x_{b}}}{P_{s}f_{c}}$$

(4) $$D_{p}\left(y_{i}\right)=\frac{\Delta \phi_{y_{b}}}{P_{s}f_{c}},$$

where $\Delta \phi_{x}$ and $\Delta \phi_{y}$ are the satellite attitude jitter in the body coordinate system, known as the pitch and the roll, as shown in Figure 1. $P_{s}N$ and $f_{c}$ are the pixel size and focal length, respectively. $D_{p}\left(x_{i}\right)$ and $D_{p}\left(y_{i}\right)$ are the image displacement in the $x_{i}$ and $y_{i}$ directions, respectively. Therefore, given that the pixel size and focal length are invariant for a specific satellite, the equation of the image displacement is the same network as the attitude jitter in Equation (2). Moreover, the image displacement reflects the image deformation directly.

Then, the image displacement formula with random parameters is used in the high-resolution remote sensing images and the distorted images are generated. In addition, original and distorted image pairs are prepared for training and evaluating our models.

### 2.2. GAN-Based Jitter Estimation

GAN was first proposed by Goodfellow et al. [31] in 2014.The idea of a GAN is to define a game between two competing networks: discriminator and generator. Generator G can capture the potential distribution of real data and output new data, while discriminator D is a binary classifier that can judge whether the input samples are real. We proposed a new GAN-based image jitter compensation network (RestoreGAN) for remote sensing images. Compared with the previous architecture, two convolution blocks with large kernels are first applied in this work, which are helpful to learn the HR features and improve network capability. Then, one stride convolution block and two residual blocks with batch normalization are introduced. The discriminator network of the GAN can be regarded as a regularization method that can force the generator network to learn essential features and mitigate overfitting to a great extent. From a theoretical perspective, the ultimate aim of the GAN architecture is to solve the following minimax problem:

(5) $$\min_{G}\max_{D}=\underset{x∼p_{r}}{E}\left[\log\left(D\left(x\right)\right)\right]-\underset{\tilde{x}∼p_{q}}{E}\left[\log\left(1-D\left(\tilde{x}\right)\right)\right],$$

where $P_{r}$ is the data distribution and $P_{g}$ is the model distribution. *E* is the expectation operator. The basic GAN structure encounters several problems, such as difficult convergence, mode collapse, and vanishing gradient, as described in [32]; therefore, a Wasserstein GAN (WGAN) [33] was proposed to improve the feasibility and efficiency. The Earth mover’s distance (Wasserstein distance) is introduced to rewrite Equation (5) as:

(6) $$\min_{G}\max_{D\in L}=\underset{x∼p_{r}}{E}\left[D\left(x\right)\right]-\underset{\tilde{x}∼p_{q}}{E}\left[D\left(\tilde{x}\right)\right]$$

where *L* is the set of 1-Lipschitz functions. By introducing Lipschitz functions, WGAN provides a gradient to push $P_{g}$ towards $P_{r}$. Then, a weight clipping strategy is utilized to enforce a Lipschitz constraint in the WGAN.

**Figure 3:**
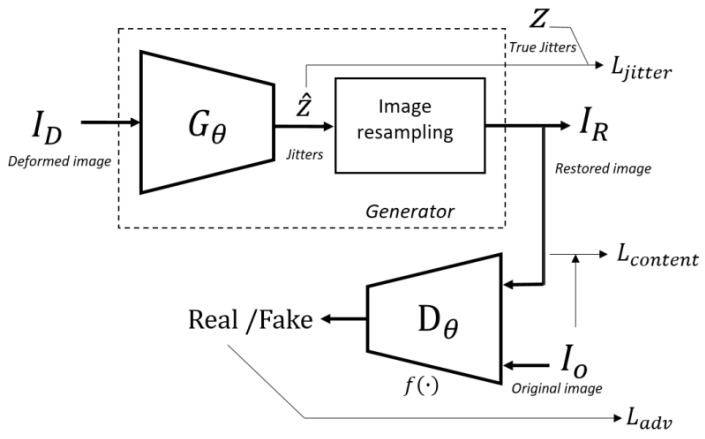
Overview of the proposed RestoreGAN.

Given the deformed remote sensing image $I_{D}$, the goal is to detect the image jitter and output the compensated image $I_{R}$. To achieve this goal, a CNN referred to as Generator $G_{\theta }$ is created. For each $I_{D}$ and network $G_{\theta }$, the $I_{R}$ is estimated. Simultaneously, during the training phase, the output of Generator $G_{\theta }$ and the original image $I_{O}$ are introduced into another CNN referred to as Discriminator $D_{\theta }$, which helps the training of $G_{\theta }$ in an adversarial manner. To generate jitter vectors simultaneously, the final layer of the $G_{\theta }$ is a fully connected layer, and the network output is the jitter vectors in two directions. Then, an image resampling model is introduced to interpolate the deformed images and retrieve the restored images. An overview of RestoreGAN is given in Figure 3.

As shown in Figure 3, the loss function of the proposed architecture is formulated as a combination of content loss, jitter loss and adversarial loss:

(7) $$L_{GAN}=L_{adv}+\lambda_{1}L_{con}+\lambda_{2}L_{jit},$$

where $\lambda_{1}$ and $\lambda_{2}$ are hyperparameters that control the weights of different losses.

#### 2.2.1. Adversarial Loss

The format of the critical function from WGAN is utilized to achieve stable, high-quality results, and to reduce the instability of the GAN training, feature matching loss [32] is implemented. Unlike the classic GAN architecture in which $G_{\theta }$ is updated based on the binary output of $D_{\theta }$ (real or fake), feature matching loss updates $G_{\theta }$ based on the internal representation of $D_{\theta }$. Let f(x) be a function that outputs an intermediate layer of the discriminator $D_{\theta }$ for a given input *x*. The adversarial loss is calculated as follows:

(8) $$L_{adv}=\frac{1}{N}\sum_{n=0}^{N}{\left(f\left(I_{o}\right)-f\left(I_{R}\right)\right)}^{2},$$

where *N* is the number of image batches.

#### 2.2.2. Content Loss

To optimize the generator towards learning contextual information of the correct images, content loss is introduced to penalize the generator by measuring the distance between the correct images and the generated images. According to [34], L1-type loss yields less blurry results than does L2-type loss. Hence, the L1 distance between the correct and generated images is utilized as the content loss, which is defined as follows:

(9) $$L_{con}=\frac{1}{N}\sum_{n=0}^{N}∥\left(I_{o}-I_{R}\right)∥.$$

#### 2.2.3. Jitter Loss

In this task, jitter loss is the Euler distance between the generated jitter vectors and the true jitter vectors. Jitter loss is introduced to help the generator find the correct direction to reduce the loss and thereby accelerate the training phase. Jitter loss is defined as follows:

(10) $$L_{jit}=\frac{1}{N}\sum_{n=0}^{N}{\left(\hat{z}-z\right)}^{2}.$$

**Figure 4:**
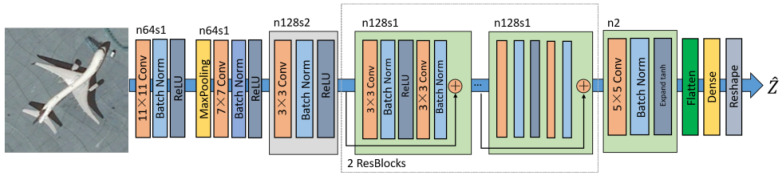
Architecture of the proposed generator in RestoreGAN.

The architecture of the discriminator in RestoreGAN is the same as that of the discriminator from DCGAN [35] and is illustrated in Figure 4. Compared with the previous architecture [36], two convolution blocks with large kernels are first applied, which has been proven [37] helpful to learn the HR features and improve network capability. Then one stride convolution block and two residual blocks with batch normalization are introduced. After that, another one stride convolution block with Expanded tanh(Et) layer is introduced due to the large amplitude of the output $\hat{Z}$. The ordinary tanh function clips the input into −1 to 1, and the Expanded tanh layer can expand the input into the range from *A* to −A by multiplying an expanded factor A.

After applying the flatten layer and reshape layer, the proposed generator outputs the two directional jitter vectors with the same length as the image height. The generated jitter is then utilized in the loss function to train RestoreGAN.

### 2.3. Image Area Selection

**Figure 5:**
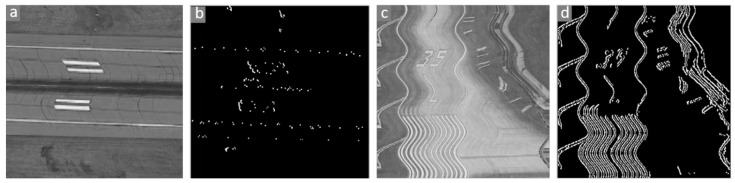
Edge detection by the Sobel operator in the vertical direction. (**a**): An example deformed image with horizontal features; (**b**): Edge detection result of (**a**); (**c**): An example deformed image with vertical features; (**d**): Edge detection result of (**d**).

In practice, when applying pretrained models to deformed remote sensing images, image patches of the same size as the model input should be selected to detect jitter and compensate the images. Considering that different scenes present different degrees of deformation and that the pixels of the same imaging lines suffer the same jitter, some image samples with obvious deformation are retrieved from the raw image to detect jitter. Because the onboard camera scans scenes in the horizontal direction, vertical features are more sensitive to jitter deformation than are horizontal features. Figure 5a,c shows two extreme situations in which jitter occurs on a horizontal road and a vertical road. In this work, we introduce a Sobel operator in the vertical direction to detect image edges, as shown in Figure 5b,d. The Sobel operator is defined as below:

(11) $$S=\left[\begin{array}{ccc} -1 & 0 & 1\\ -2 & 0 & 2\\ -1 & 0 & 1 \end{array}\right]$$

Thus, the image deformation metric (IDM) is proposed to evaluate the deformation degree of image patches. The IDM formula is as follows:

(12) $$IDM=\frac{1}{WH}\sum_{x=0}^{W}\sum_{y=0}^{H}\left(Cov\left(I_{R},S\right)\right),$$

where Cov represents the convolution operation and *W* and *H* are the width and height of the images. Image patches with high IDM are selected as inputs of the proposed method, and the raw image is then corrected by image interpolation with combined jitter.

## 3. Experimental Results and Discussion

### 3.1. Data Preparation

In this task, three popular datasets, PatternNet [21], UC Merced [38] and WHU-RS19 [39], are adopted to validate the proposed method. Moreover, a deformed image from the Yaogan-26 satellite [7] is introduced to verify the proposed method.

The PatternNet dataset was collected by Zhou et al. from HR imagery and contains 38 classes with 800 images per class. Each image in the dataset is 256 × 256 pixels with RGB channels. The images were manually extracted from large images from the USGS National Map Urban Area Imagery collection for various urban areas around the country. The pixel resolution of this public domain imagery is one foot.

The UC Merced Land Use dataset, released in 2010, contains 21 classes with 100 images per class. The images were manually extracted from large images from the USGS National Map Urban Area Imagery collection for various urban areas around the country.

The WHU-RS19 dataset was extracted from a set of satellite images exported from Google Earth with spatial resolution up to 0.5 m and spectral bands of red, green, and blue. The dataset is composed of 19 classes of different scenes with 50 images per class. Each image in the dataset is 600 × 600 pixels.

The Yaogan-26 satellite launched by China in 2014 is a HR optical satellite that orbits synchronously against the Sun at an altitude of 490 km. Due to various attitude maneuvers, the remote sensing images suffer from low-frequency satellite jitter. In the Yaogan-26 satellite platform, the high-frequency angular displacement sensor can measure platform jitter in a frequency range of 0.2∼450 Hz and output high-frequency attitude jitter data, which provides a good method for the on-ground compensation of the jitter in an image. The deformed image and the image corrected by onboard sensors are introduced to validate our methods.

To prepare the dataset to train and validate the proposed methods, the RGB images from each dataset are transformed into gray images, and each image is resized 256 × 256 pixels. Equation (2) with random parameters is then applied to the images to generate deformed and correct image pairs. Then, the deformed images are resized to 128 × 128 pixels to accelerate the training process. To verify the generality of the proposed RestoreGAN, only five image classes from the PatternNet dataset with palpable edge features are selected for training. The training classes are ’solarpanel’, ’freeway’, ’parkingspace’, ’runway’, and ’overpass’. Additionally, one-fourth of each class in the training data was utilized for validation to evaluate the training performance and to monitor overfitting in the training phase.

### 3.2. Training Details

We implemented all our models using the Tensorflow deep learning framework. All methods were trained in the Python environment with a i7 CPU with 16 GB RAM and a GeForce GTX 1060 GPU with 6GB RAM. For optimization, we performed gradient descent at each time point for $G_{\theta }$ and $D_{\theta }$ using Adam as a solver. The learning rate was initially $1\times 10^{-4}$ for both the generator and discriminator, and the learning rate decayed every 10 iterations to accelerate the training process. The batch size of the training process was five due to the memory limitations of the GPU.

### 3.3. Results on Different Frequencies and Amplitudes

**Figure 6:**
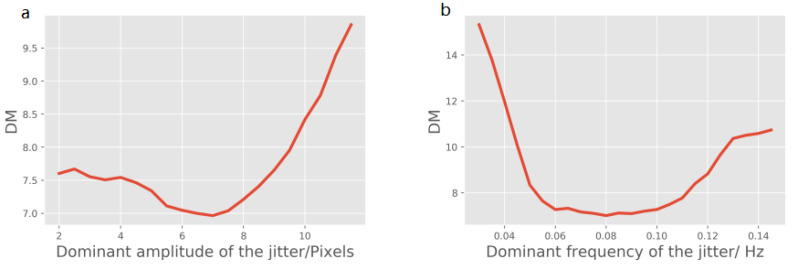
Jitter test results with different dominant frequencies and amplitudes on pretrained RestoreGAN. (**a**): Loss of RestoreGAN for deformed images with different dominant amplitudes; (**b**): Loss of RestoreGAN for deformed images with different dominant frequencies.

**Figure 7:**
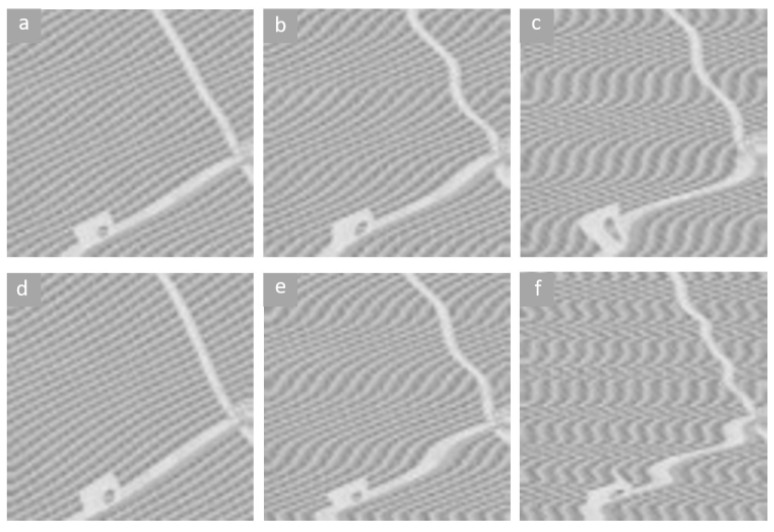
Deformation results of Equation (2) for different dominant frequencies and dominant amplitudes. (**a**): Amplitude = 2 pixels; (**b**): Amplitude = 6 pixels; (**c**): Amplitude = 11 pixels; (**d**): Frequency = 0.03 Hz; (**e**): Frequency = 0.07 Hz; (**f**): Frequency = 0.13 Hz.

According to the jitter results from the ZY-3 satellite [40], Yaogan-3 satellite [7] and ALOS satellite [3], the image jitter can be considered to be a combination of a sinusoidal function with the dominant frequency and several noisy sine curves with different frequencies. The dominant frequency and amplitude vary by satellites and image resolution. To verify the generalization ability of the proposed method, the restoration results for the jitter of different dominant amplitudes and frequencies are illustrated. Additionally, the deformation metric (DM) is proposed to evaluate the restoration results. The DM is similar to the RestoreGAN loss from Equation (5) and is defined as follows:

(13) $$DM=100L_{con}+L_{jit},$$

where the definition of $L_{con}$ and $L_{jit}$ in Equation (13) are the same as those in Equation (5). The lower the DM is, the better the compensation results that can be acquired. Figure 6a shows that the best performance is achieved for a dominant amplitude ranging from 5 to 8 pixels. When the amplitude of the jitter is 10 pixels or more, the proposed method loses the ability to compensate the deformed images. In Figure 7, deformed images with dominant amplitudes of 2 pixels, 6 pixels, and 11 pixels are illustrated. Due to the input image size of 128 pixels, the deformation in Figure 7c is excessively severe and can be treated as an anomalous situation. Similarly, the dominant frequency with DM is illustrated in Figure 6b, and RestoreGAN shows better performance in the frequency range from 0.06 Hz to 0.10 Hz. Deformed images with dominant frequencies of 0.03 Hz, 0.07 Hz and 0.13 Hz are shown in Figure 7d–f, respectively. In Figure 7d, the deformation with low frequency looks similar to the natural curves from the other classes, which will confuse the proposed model and result in an output of near-zero jitter results. In other words, RestoreGAN may ignore the deformation when the frequency is too low. On the other hand, Figure 7c illustrates that deformation with high frequency can also be treated as an anomalous situation. Therefore, in terms of jitter deformation due to reasonable satellite vibration and platform controller bias, RestoreGAN shows good capacity to detect jitter and correct the image.

### 3.4. Image Restoration Experiments

In this experiment, the proposed RestoreGAN is compared with UnrollingCNN [37], GenCNN, and ContGAN. UnrollingCNN corrects the motion distortion caused by the rolling shutter effect of camera motion. UnrollingCNN achieves good compensation effects by using row and column kernel banks. In this work, the architecture of UnrollingCNN from [37] is utilized to generate image jitter, and the image resampling model in Figure 3 is adopted to compensate the images. GenCNN is another CNN model that adapts only the $G_{\theta }$ from RestoreGAN to correct images. The loss function of GenCNN is the same as that in Equation (7), while $L_{adv}$ is set to 0 to remove the influence of the discriminator. It is worth mentioning that the GenCNN is the same network as the IJC-Net proposed in the previous work [36]. ContGAN is a GAN that considers only the content loss during the training phase. ContGAN has the same architecture as RestoreGAN, with $\lambda_{2}$ in Equation (7) equal to 0. All the methods are trained on the same images from five classes of the PatternNet dataset, and the parameters of the backpropagation process are also the same as those of RestoreGAN. The effectiveness of different compensation methods is evaluated based on the image restoration results.

**Figure 8:**
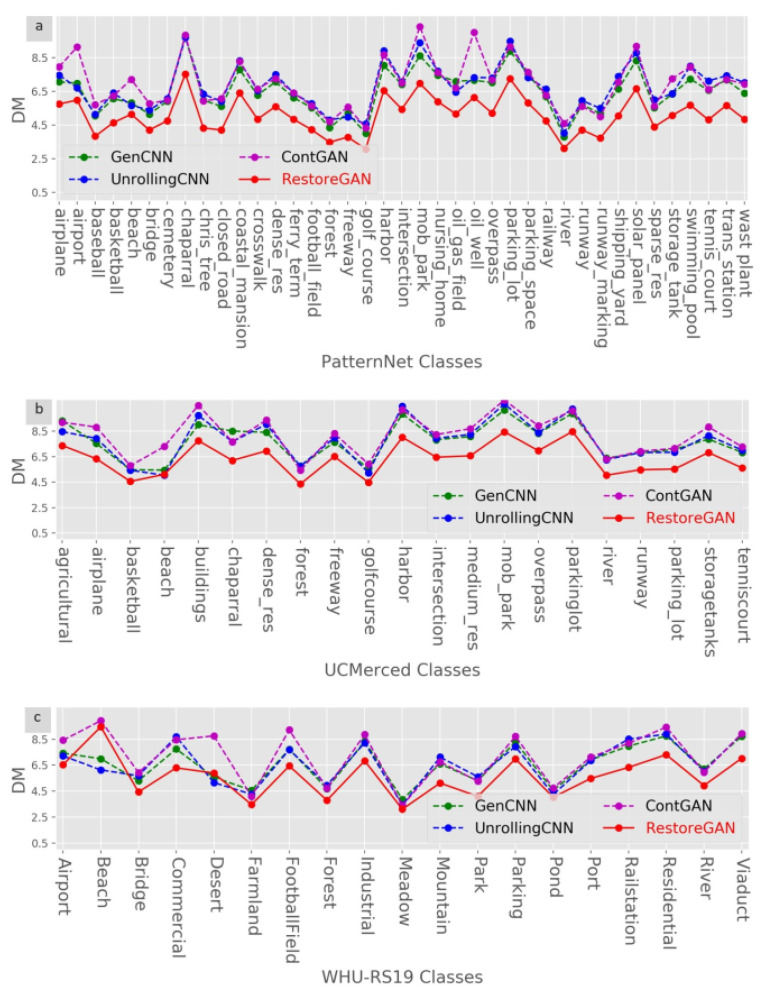
Restoration results of different methods on the three datasets. (**a**): DM results on the PatternNet dataset; (**b**): DM results on the UCMerced dataset; (**c**): DM results on the WHU-RS19 dataset.

Figure 8 shows the restoration results of the different methods on the three datasets. Clearly, the proposed RestoreGAN has the best DM results in almost all classes of all the datasets. The experimental results provide solid evidence that RestoreGAN generally has superior deformed image restoration performance compared with other methods. Moreover, GenCNN and ContGAN achieve worse results for most classes, which means that jitter loss makes a greater contribution than does adversarial loss in the training phase. The results of GenCNN and UnrollingCNN illustrate the effectiveness and necessity of the GAN structure with adversarial loss in the training phase.

**Figure 9:**
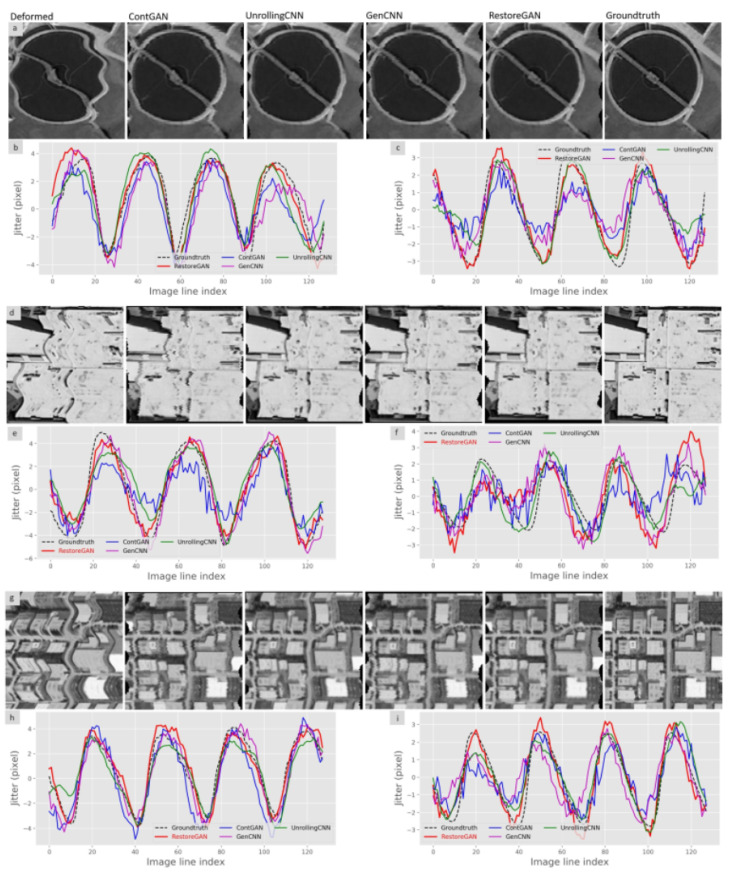
Restoration results of different models on the three datasets. (**a**): Restoration results on the wastewater treatment plant image of the PatternNet dataset; (**b**): Corresponding jitter in the x direction; (**c**): Corresponding jitter in the y direction; (**d**): Restoration results on the building image of the UCMerced dataset; (**e**): Corresponding jitter in the x direction; (**f**): Corresponding jitter in the y direction; (**g**): Restoration results on the industrial image of the WH-RSU19 dataset; (**h**): Corresponding jitter in the x direction; (**i**): Corresponding jitter in the y direction.

Figure 9 presents deformed images from different datasets that are corrected by the different methods compared to the ground truth. Figure 9a shows that the deformed circular features from the wastewater treatment plant image are learned and corrected by the proposed method. Compared with the other methods, the proposed method can largely correct the slight twist and odd inflections in the plant edges. In Figure 9b,c, the restored jitter in the x and y directions is plotted against the ground truth. The estimated jitter indicates that the proposed method generates the best jitter among all the methods, especially in the peak area of the jitter. The buildings in Figure 9d illustrate the excellent performance of the proposed method on human-made scenery with many straight lines. The industrial image from the WHU-RS19 dataset in Figure 9g shows that the proposed method is able to rectify the deformed features from images with resolution lower than that of the training data. The jitter detection results in Figure 9 also illustrate the generally better performance in the x direction compared with the y direction because the image deformation caused by satellite jitter is more noticeable in the x direction in most cases.

**Figure 10:**
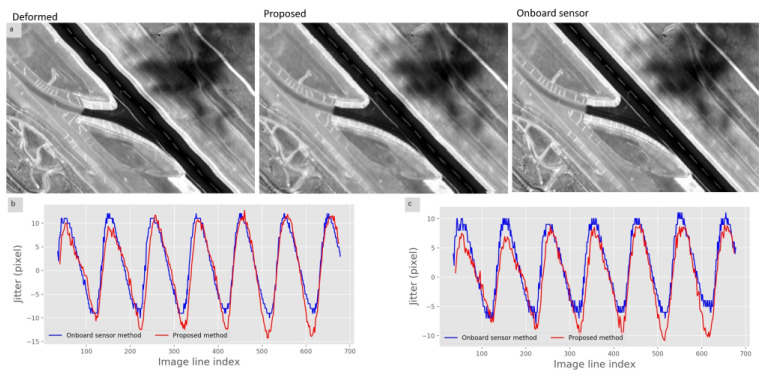
Restoration results on the Yaogan-26 satellite image dataset. (**a**): Restoration results on the Yaogan-26 satellite airport image. From left to right: Raw deformed image; Image restored by RestoreGAN; Image restored by onboard sensors; (**b**): Jitter in the x direction for the two methods; (**c**): Jitters in the y direction for the two methods.

Figure 10 shows the restoration results on the distorted Yaogan-26 high-resolution images affected by real attitude jitter. Considering the large size of the raw image, the IDM metric from Equation (12) is introduced to retrieve image patches from the raw image. Then, the jitter in each patch is detected by RestoreGAN and the jitter components of the same lines are combined. Finally, the image resampling model from Figure 3 is utilized to correct the raw image. The airport runways in Figure 10 demonstrate that the palpable image deformation can be detected and rectified successfully. Considering the restoration bandwidth explained in Figure 6, some low-frequency deformation remains in the restored image compared with the onboard sensor result. Moreover, the jitter curve obtained for the Yaogan-26 image is similar to the jitter generated from the training images, which confirms the good performance of the proposed method.

To further verify the validity of the proposed method, a pretrained ResNet50 deep learning model from [21] is established to complete the image retrieval task. The test dataset created from PatternNet is utilized, and five metrics, P@5, P@10, P@50, P@100 and mAP, are introduced to evaluate the performance. These commonly used performance metrics, means average precision (mAP) and precision at *k* (P@k where *k* is the number of retrieved images) are the averaged values over all the queries.The detailed definition of the metrics can be found in [21]. The image retrieval results are shown in Table 1, which shows that after employing the image restoration methods, the image retrieval results are substantially improved in terms of all metrics, and the proposed method achieves the best performance. Considering that satellite jitter disturbs the edge features and essential textures, the image deformation could confuse the deep-learning-based image retrieval methods and increase the retrieval error rate. The results in Table 1 demonstrate the necessity and effectiveness of jitter compensation in image retrieval tasks.

## 4. Conclusions

In this paper, the usefulness and effectiveness of GAN for HR remote sensing image restoration is explored. Based on improved GAN, the proposed method has been trained and evaluated on distorted HR remote sensing image datasets with simulated jitter vectors. The experimental results on three public datasets indicate that the proposed network architecture achieves the highest DM and best performance on most of the restored images. The image retrieval results demonstrate the necessity and effectiveness of our method in image retrieval tasks. Furthermore, the Yaogan-26 image compensation results illustrate that the deformed images can be corrected without any auxiliary sensor data, which are usually hard to obtain. In conclusion, the proposed RestoreGAN reveals the huge potential of GAN-based methods for the analysis of attitude jitter in remote sensing images. To increase the validity of this work, several aspects remain for further improvement. The first is that we expect to use fewer samples to learn better features, which is meaningful in remote sensing images. Secondly, it is normally a reasonable assumption for satellite HR images that the attitude jitter in the yaw direction is much less noticeable than that in the other two directions. However, we should also pay attention to the correction of the yaw direction jitter in remote sensing images. Moreover, the present methodology can be independently implemented with conventional methodologies, which will be a future research direction.

## Figures and Tables

**Table 1:** Image retrieval results by ResNet50 on deformed and restored images from PatternNet.

Image Type	*P* @ 5	*P* @ 10	*P* @ 50	*P* @ 100	*mAP*
Deformed	0.4263	0.41736	0.3947	0.381	0.2495
UnrollingCNN	0.6231	0.6089	0.5925	0.5726	0.3901
GenCNN	0.6157	0.6042	0.5691	0.5438	0.3622
ContGAN	0.6105	0.6079	0.5685	0.5448	0.3580
RestoreGAN	0.6979	0.6926	0.6575	0.6359	0.4180

## Data Availability

Not applicable.
